# Synergistic effects of LFchimera and antibiotic against planktonic and biofilm form of *Aggregatibacter actinomycetemcomitans*

**DOI:** 10.1371/journal.pone.0217205

**Published:** 2019-07-22

**Authors:** Marie Rossini Carmela T. Lachica, Chitchanok Anutrakunchai, Saengsome Prajaneh, Kamran Nazmi, Jan G. M. Bolscher, Suwimol Taweechaisupapong

**Affiliations:** 1 Biofilm Research Group, Faculty of Dentistry, Khon Kaen University, Khon Kaen, Thailand; 2 Department of Periodontology, Faculty of Dentistry, Khon Kaen University, Khon Kaen, Thailand; 3 Department of Oral Biochemistry, Academic Centre for Dentistry Amsterdam (ACTA), University of Amsterdam and VU University Amsterdam, Amsterdam, The Netherlands; Laurentian, CANADA

## Abstract

Adjunctive use of antibiotics in periodontal treatment have limitations and disadvantages including bacterial resistance. Antimicrobial peptides (AMPs) are potential new agents that can combat bacterial infection. In this study, antimicrobial activity of different concentrations of conventional antibiotics minocycline (MH), doxycycline (DOX), and antimicrobial peptides LL-37, LL-31, Lactoferrin chimera (LFchimera) and Innate Defense Regulator Peptide 1018 (IDR-1018) against *Aggregatibacter actinomycetemcomitans* ATCC 43718 were determined using colony culturing assay. Subsequently, *in vitro* activity of the most effective drug and peptide combination was evaluated by checkerboard technique. Impact of the drug and peptide co-administration on biofilm at different stages, i.e., during adhesion and 1-day old biofilm was compared to each of the agents used alone. Results revealed that the killing effects of all AMPs range from 13–100%. In contrast, MH and DOX at 1 and 5 μM showed no killing activity and instead stimulated growth of bacteria. DOX has better killing activity than MH. LFchimera displayed the strongest killing amongst the peptides. Checkerboard technique revealed that combining DOX and LFchimera yielded synergism. Confocal laser scanning microscopy further showed that the combination of DOX and LFchimera caused significant reduction of bacterial adhesion and reduction of biomass, average biofilm thickness and substratum biofilm coverage of 1-day old biofilm compared to DOX and LFchimera alone. In conclusion, LFchimera alone and in combination with DOX exhibited strong antibacterial and anti-biofilm property against *A*. *actinomycetemcomitans*. The findings suggest that LFchimera should be considered for development as a new potential therapeutic agent that may be used as an adjunctive treatment for periodontitis.

## Introduction

Periodontitis is a common disease of the oral cavity that affects millions of people world-wide. According to the World Health Organization, 10–15% of the world population suffers from severe periodontitis [1]. Though millions of people are affected by the disease, there continues to be no ideal chemotherapeutic agent for the treatment of periodontitis. Up to date, the quest to develop an ideal chemotherapeutic agent to help improve the treatment of periodontal disease continues.

*Aggregatibacter actinomycetemcomitans* is one of the most common bacteria involved in periodontal disease. It is associated with what was previously called localized aggressive form of periodontitis (LAP) that usually affects the younger population [2, 3]. LAP causes rapid attachment and bone loss [4, 5] and has shown to adversely affect the psychology, function and aesthetic of an individual [6]. Some forms of periodontal disease like LAP or refractory periodontitis need adjunctive antibiotic in its course of treatment. Antibiotic groups that have been thoroughly studied for treatment of periodontal disease include tetracycline, minocycline (MH), doxycycline (DOX), clindamycin, amoxicillin, ampicillin, metronidazole and erythromycin [7]. Of these antibiotics, only four of them are available for local drug delivery: tetracycline, DOX, MH and metronidazole [8, 9]. Though these antibiotics have been effective adjuncts to periodontal treatment, they also carry certain limitations and disadvantages including bacterial resistance. Previous studies reported resistance of periodontopathic bacteria isolated in patients with chronic periodontitis to several antimicrobial agents commonly used in anti-infective periodontal therapy [10, 11]. With the increasing development of antibiotic resistance, it is invaluable to search for novel anti-infective agents.

Antimicrobial peptides (AMPs) are conserved biomolecules in all organisms and are considered a major component of the innate immune response. They have received special attention as a possible solution that can combat antibiotic-resistant bacterial strains. AMPs are attractive candidates for clinical development because of their selectivity, speed of action and low potential for resistance development. There are limited studies however on the use of AMPs against periodontopathic bacteria. This study will employ 4 different AMPs: LL-37 is the AMP derived from the only cathelicidin in humans [12]; LL-31 is a truncated variant of LL-37 missing the six residues at the C-terminus; Lactoferrin chimera (LFchimera) is a heterodimeric peptide containing lactoferrampin (LFampin265–284) and a part of lactoferricin (LFcin17–30) from parent protein bovine lactoferrin [13] and Innate defense regulator peptide—1018 (IDR-1018), a synthetic derivative of endogenous cationic host defense peptides [14].

Current literature would show numerous studies of AMP on oral pathogens with the human cathelicidin-derived peptide, LL-37. However, there is no report on the killing activities of LL-31, LFchimera, and IDR-1018 against periodontopathic bacteria compared to MH and DOX. There is also no report of possible synergistic effect of drug and peptide combination. This study aims to search for the most effective peptide and antibiotic against *A*. *actinomycetemcomitans* ATCC 43718. It also aims to determine possible synergism of the best peptide and drug combination on planktonic form and different stages of *A*. *actinomycetemcomitans* biofilm formation.

## Materials and methods

### Antimicrobial peptides and antibiotics

The peptides used in this study include LL-37, its truncated variant LL-31, LFchimera and IDR-1018. All peptides were synthesized using Fmoc-protected amino acids (Orpegen Pharma GmbH, Heidelberg, Germany) with a Syro II peptide synthesizer (Biotage, Uppsala, Sweden) and purified with an Ultimate 3000 RP-HPLC (Thermo Scientific, MA) to a purity of at least 95% as previously described [15]. The authenticity of the peptides was confirmed by Matrix-Assisted Laser Desorption/Ionization Time-of-Flight Mass Spectrometry (MALDI-TOF MS) on a Microflex LRF mass spectrometer equipped with an additional gridless reflection (Bruker Daltonik, Bremen, Germany) as described previously [15]. Amino acid sequences and characteristics of the peptides investigated are shown in Table 1. The antibiotics used were minocycline hydrochloride (Sigma-Aldrich, St. Louis MO) and doxycycline (Sigma-Aldrich, St. Louis MO); two drugs commonly used as adjunctive treatment for periodontitis.

**Table 1:** Sequences and characteristics of the peptides investigated.

Peptide	Sequence	Mol. Wt.	Net Charge^a^
LL-37	LLGDFFRKSKEKIGKEFKRIVQRIKDFLRNLVPRTES	4493	6+
LL-31	LLGDFFRKSKEKIGKEFKRIVQRIKDFLRNL	3821	6+
LFchimera	FKCRRWQWRMKKLG—K DLIWKLLSKAQEKFGKNKSR	4422	12+
IDR-1018	VRLIVAVRIWRR-NH_2_	1536	4+

^a^Net positive charge at neutral pH

### Bacterial strain and growth condition

*A*. *actinomycetemcomitans* ATCC 43718, was maintained on tryptic soy serum bacitracin vancomycin (TSBV) agar. A single colony initially grown on TSBV agar was inoculated into Todd Hewitt broth (THB), incubated at 37°C, 5% CO_2_ for 16–18 h and used as inoculum in the antimicrobial assay.

### Antimicrobial assay of planktonic bacteria

Colony culturing assay was used to determine killing activity of various concentrations of MH, DOX, LL-37, LL-31, LFchimera and IDR-1018 as described previously [16]. Bacterial cells were washed and re-suspended to a final inoculum of ~ 1 x 10^6^ CFU/ml in 1 mM potassium phosphate buffer (PPB), pH 7.0. The bacterial suspension was then added to the antimicrobial agents to reach a final concentration of 1, 5, 10, 20, 40 and 50 μM and incubated at 37°C, 5% CO_2_ for 1 h. A bacterial suspension without antimicrobial agents served as a control. After 1 h of incubation, the mixtures were serially diluted in physiological concentration of saline and plated in triplicate on nutrient agar (NA). The plates were incubated at 37°C, 5% CO_2_ for 48 h and colonies were counted thereafter. The percentage killing effects of each agent was calculated using the formula [1 - (CFU sample/CFU control)] x 100%. Each assay was performed on three separate occasions, with triplicate determinations each time.

### Drug and peptide combination against planktonic bacteria

The drug that demonstrated the best killing activity from the previous experiment was selected to analyze in combination with the most effective peptide to determine possible synergistic effect on the killing activity on *A*. *actinomycetemcomitans*. The interaction between the antimicrobial agents was determined through checkerboard broth microdilution technique with some modifications [17]. The half maximal inhibitory concentration (IC_50_) of the antibiotic and peptides was serially diluted in 1 mM PPB to concentrations range from 1 to 1/8 times of IC_50_. The bacterial suspension (~ 1x 10^6^ CFU/ml) was then added to equal volume of the combined antimicrobial agents on the 96-well plate and was incubated at 37°C, 5% CO_2_ for 1 h. A bacterial suspension in PPB without antimicrobial agent, served as a control. After which, the mixtures were serially diluted in physiological concentration of saline and plated in triplicate on NA. The plates were incubated at 37°C, 5% CO_2_ for 48 h and colonies counted thereafter. Three independent experiments were performed on separate occasions. Fractional inhibitory concentration index (FICI) was calculated using the formula: FICI = (IC_50AP_ / IC_50A_) + (IC_50PA_ / IC_50P_) where IC_50AP_ is the IC_50_ of the antibiotic in combination, IC_50A_ is the IC_50_ of the antibiotic alone, IC_50PA_ is the IC_50_ of the peptide in combination and IC_50P_ is the IC_50_ of the peptide alone. Synergism was defined by FICI value ≤ 0.5, no interaction by 0.5 < FICI ≤ 4.0 and antagonism by FICI > 4.0 [18].

### Effect of drug and peptide alone and in combination on adhesion

The drug and peptide concentration that demonstrated the best synergism from the previous experiment was further studied for their effects on adhesion of *A*. *actinomycetemcomitans* compared to the drug and peptide alone using Amsterdam Active Attachment (AAA) model [19]. The AAA model consists of a stainless steel lid with clamps that contain 12 mm diameter round glass coverslips which were used as substratum for adhesion of *A*. *actinomycetemcomitans*. The lid fits onto standard polystyrene 24-well plates (Corning, USA). After assembling the lid and glass coverslips, the model was autoclaved.

Overnight culture of bacteria was re-suspended in 1 mM PPB and adjusted to give an optical density at 540 nm (OD_540_) of 0.5. The bacterial suspension was then added to equal volume of the antimicrobial agents. A well with only 1 mM PPB served as control. To allow adhesion on each glass coverslips, the plate was covered with a sterile stainless-steel lid containing the glass coverslips and incubated at 37°C, 5% CO_2_ for 30 min. The glass coverslips were then washed twice and sessile cells were stained using Live/Dead BacLight Bacterial Viability kit (Molecular Probes Inc., USA) then viewed using a confocal laser scanning microscope (CLSM, Zeiss LSM 800, Zeiss, Germany). The quantitative estimation of bacterial cells was determined from 20 random areas of 2 coverslips for each sample using the ZEN image analysis wizard software (Zeiss, Germany).

### Effect of drug and peptide alone and in combination on 1-day old biofilm matrix

The drug and peptide concentration that demonstrated the best synergism from the result of the previous experiment was further studied for their effects on 1-day old *A*. *actinomycetemcomitans* biofilm matrix compared to the drug and peptide alone as previously described [16]. Overnight culture of bacteria was re-suspended in fresh THB media to give an OD_540_ of 0.9 and transferred to each well of the 24-well plate. The plate was covered with the AAA model containing the glass coverslips and incubated at 37°C, 5% CO_2_ for 3 h to allow for bacterial adhesion. After 3 h, the lid was transferred to new 24-well plate containing fresh media and incubated for an additional 21 h. The 1-day old biofilms were placed on wells containing either 1 mM PPB (control) or antimicrobial agents for 15 min. The coverslips were then washed twice and fixed with 1% glutaraldehyde for 3 h at 4°C. Next, the extracellular polymeric substance of the biofilm was stained with fluorescein isothiocynate-concanavalin A (FITC-ConA) for 15 min. The glass coverslips were examined using CLSM. A total of 11 randomly selected areas of the coverslips were examined for each sample. Z-stacks images of the biofilm were collected at 0.5 μm interval through the biofilm. Data on the structure of the biofilm was collected and 3-dimensional images were reconstructed using the Zen image software. Biomass of extracellular polysaccharides [20], substratum coverage and average biofilm thickness was analyzed using COMSTAT 2.1 software [21].

### Statistical analysis

The comparison between each antimicrobial agent was analyzed using MedCalc statistical software, version 13.0.6 (Ostend, Belgium). Comparison between the two antimicrobial drugs were done using Mann-Whitney test. Kruskal-Wallis analysis and Conover’s multiple comparison tests were carried out for comparison between antimicrobial agents. The level required for statistical significance was *P* < 0.05.

## Results

### Susceptibility of *A*. *actinomycetemcomitans* in planktonic form to antimicrobial agents

**Fig 1:**
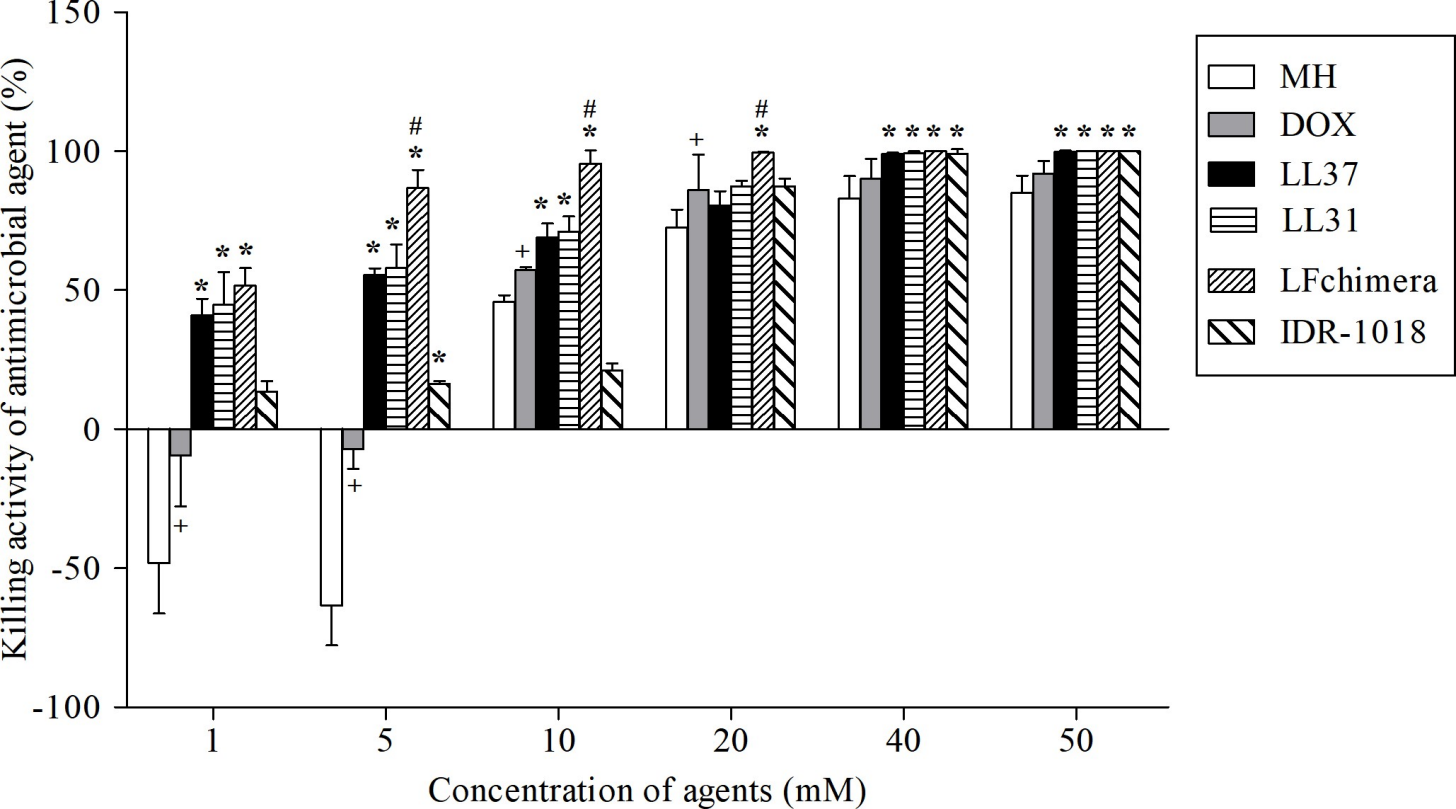
Killing activity of antimicrobial agent. Effect of the different concentrations of the 2 antibiotics and the 4 peptides against *A*. *actinomycetemcomitans*. Bacterial suspensions were incubated with 1, 5, 10, 20, 40 and 50 μM of each agent and processed as described in materials and methods. Data are the mean ± SD of triplicates from three independent experiments. *P < 0.01 compared with both MH and DOX, ^#^*P* < 0.01 compared with other peptides, ^+^*P* < 0.05 compared with MH.

The killing activity of each agent was determined by colony culturing assay (Fig 1). The results revealed that the killing effects of all agents on *A*. *actinomycetemcomitans* appeared to be dose-related. The antimicrobial activities of all AMPs range from 13–100%. At the highest concentration used (50 μM), all peptides displayed 100% killing. In contrast, MH and DOX at low concentrations (1 and 5 μM) not only showed no killing activity but also stimulated growth of the bacteria. All concentrations of LL-31 and LFchimera exhibited significantly higher antimicrobial activities than both MH and DOX (*P* < 0.01). Between the two drugs, DOX exhibited higher killing activity than MH (*P* < 0.05). The IC_50_ value of DOX determined using linear regression was 11 μM. Among all peptides tested, all concentrations of LFchimera possessed the strongest killing activity towards *A*. *actinomycetemcomitans*. The average killing activity of 1 μM LFchimera was 51.63 ± 9.08%, hence its IC_50_ was set at 1 μM.

The IC_50_ values of DOX and LFchimera were used to explore possible synergism between the two agents by the broth microdilution checkerboard technique. The concentrations of DOX and LFchimera that yielded synergism with their corresponding percent killing activity are shown in Table 2. All combinations with FICI values ≤ 0.5 have higher killing activity compared to the concentration of agents alone. The most synergistic combination of 1.4 μM DOX and 0.125 μM LFchimera with FICI value of 0.25 has a killing activity 7.2 times higher than 1.4 μM DOX alone and 2.5 times higher than 0.125 μM LFchimera alone. Furthermore, the combination has 66% killing activity even when the concentration of both agents was reduced 8 fold from the IC_50_ value.

**Table 2:** FICI values and percent killing activity of synergistic combinations of DOX and LFchimera against *A*. *Actinomycetemcomitans*.

Concentration of DOX / LFchimera in combination (μM)	FICI	Killing Activity (%)
1.4 / 0.125	0.25	66
1.4 / 0.25	0.38	62
2.8 / 0.125	0.38	76

### Synergistic effects of DOX and LFchimera on adhesion and 1-day old biofilm matrix of *A*. *actinomycetemcomitans*

**Fig 2:**
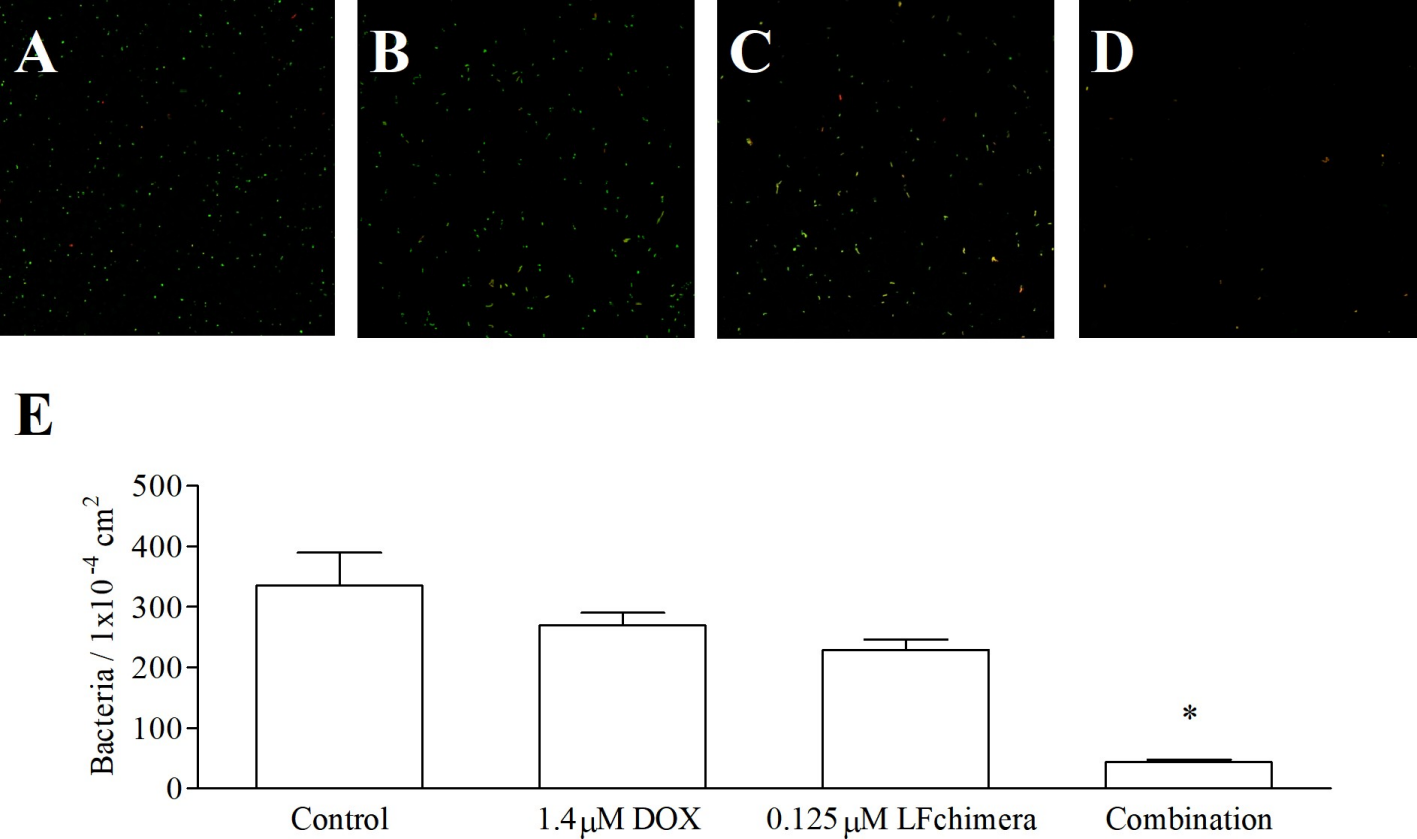
Effect of antimicrobial agent on adhesion state of biofilm-forming. Confocal laser scanning micrographs of *A*. *actinomycetemcomitans* attached cells on glass coverslips after 30 min incubation in 1mM PPB (A), 1.4 μM DOX (B), 0.125 μM LFchimera (C) and combination of 1.4 μM DOX and 0.125 μM LFchimera (D). Bacteria were stained with LIVE/DEAD BacLight Bacterial Viability kit. Green color indicates live bacteria and red color indicates dead bacteria. Images were viewed at 630x magnification. (E) Bacterial count from 20 random areas of 2 coverslips for each sample. Data presented are the mean ± SD of adherent bacteria. *P < 0.01 compared with control and other agents.

Confocal laser scanning micrographs confirm differences in the effect of the most synergistic combination on bacterial adhesion compared to agents alone (Fig 2). The images of the control coverslip (Fig 2A) and 1.4 μM DOX alone treated coverslip (Fig 2B) show a greater amount of green fluorescence indicating more adherence of alive bacteria. On the contrary, a clear decrease in green fluorescence with an increased red fluorescence is seen both on 0.125 μM LFchimera treated (Fig 2C) and on the combination treated (Fig 2D) coverslips. Quantification of bacterial adherence using the Zen image analysis wizard software showed that 1.4 μM DOX and 0.125 μM LFchimera alone and in combination are able to decrease the number of adherent bacteria compared to control. Furthermore, the combination of 1.4 μM DOX and 0.125 μM LFchimera has significantly decreased bacterial adhesion by 87% less than control, 84% less than that of 1.4 μM DOX alone and 81% less than 0.125 μM LFchimera alone (Fig 2E).

**Fig 3:**
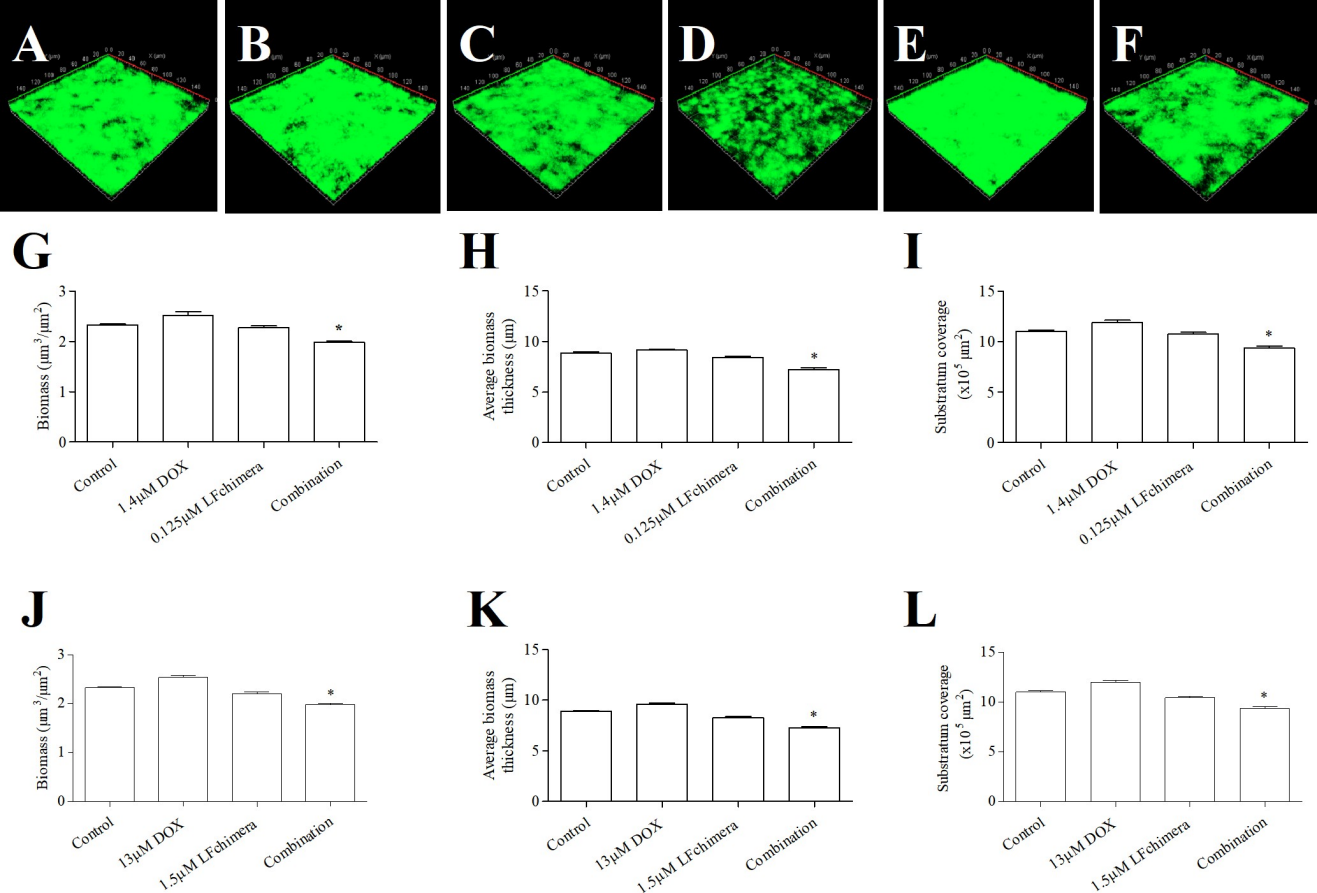
Effect of antimicrobial agent on biofilm formation. CLSM 3-D reconstruction of 1-day old *A*. *actinomycemcomitans* ATCC 43718 biofilm after 15 min exposure to 1 mM PPB (A), 1.4 μM DOX (B), 0.125 μM LFchimera (C), combination of 1.4 μM DOX and 0.125 μM LFchimera (D), 13 μM DOX (E) and 1.5 μM LFchimera (F). The biofilms were stained with FITC-ConA. Green color indicates exopolysaccharide of biofilm matrix. CLSM-COMSTAT analysis comparing the effect of 1 mM PPB, 1.4 μM DOX, 0.125 μM LFchimera and the combination on biomass (G), average biomass thickness (H) and substratum coverage (I). CLSM-COMSTAT analysis comparing the effect of 1mM PPB, 13 μM DOX, 1.5 μM LFchimera and the combination on biomass (J), average biomass thickness (K) and substratum coverage (L). Values are means ± SD from 2 independent experiments. *P < 0.01 compared with control and other agents.

CLSM images and COMSTAT analysis of 1-day old *A*. *actinomycetemcomitans* biofilm after exposure to the tested agents are shown in Fig 3. The difference among the biomass of the control (Fig 3A), 1.4 μM DOX (Fig 3B) and 0.125 μM LFchimera (Fig 3C) groups was rather indistinct. On the contrary, the biofilm that underwent treatment of the combination of 1.4 μM DOX and 0.125 μM LFchimera (Fig 3D) showed decrease green fluorescence with a striking increase in black areas indicative of areas with no biofilm present. Areas with decreased biofilm thickness are also more notable from the image. COMSTAT analysis for biofilm quantification show that among the 4 groups initially tested, the combined drug and peptide have significantly less biomass (Fig 3G), significantly thinner biofilm in terms of average biofilm thickness (Fig 3H) and significantly less biofilm surface coverage (Fig 3I) (*P* < 0.01).

The combination of 1.4 μM DOX and 0.125 μM LFchimera was further compared to 13 μM DOX and 1.5 μM LFchimera which has 66% killing activity equal to that of the combination based on the results from the checkerboard experiment. Even when compared to a higher concentration of DOX and LFchimera (Fig 3E and 3F), the combination of 1.4 μM DOX and 0.125 μM LFchimera demonstrated much higher effect on the 1-day old biofilm matrix (Fig 3D). Confocal laser micrographs reveal that comparing to control (Fig 3A), 13 μM DOX (Fig 3E) shows definite increase in abundance of fluorescence. LFchimera at 1.5 μM shows relatively more black areas with seemingly thinner biofilm and decreased patches of green fluorescence over the entire surface area (Fig 3F). There is noticeable decrease of fluorescence and disruption of the biofilm when compared to control.

COMSTAT analysis of the control, 13 μM DOX, 1.5 μM LFchimera and combination show that among all the groups, the combination had the most significant decrease in biomass (Fig 3J), average biofilm thickness (Fig 3K) and demonstrated a significantly less surface biofilm coverage (Fig 3L) amongst all groups (*P* < 0.01). It is notable that 13 μM DOX significantly has the thickest average biofilm thickness, the most abundant biomass and broadest surface area covered by the biofilm (*P* < 0.01). The 1.5 μM LFchimera treated coverslips demonstrated a significant decrease in all measured variables when compared to control (*P* < 0.01).

## Discussion

Periodontitis is a debilitating condition caused by specific bacterial complexes in the dental plaque. *A*. *actinomycetemcomitans* is a periodontopathic bacteria considered to have very high pathogenicity [22]. Amongst all periodontal bacteria, *A*. *actinomycetemcomitans* was chosen for this study because of its virulence, difficulty of treatmeant [23] and ability to resist antibiotics [24, 25].

The antimicrobial ability of four AMPs were investigated since there have been previous reports on their bactericidal activity on other gram-negative bacteria which is similar to periodontopathic bacteria [13, 26–32]. The results of this study confirmed the potency of the four peptides towards another gram-negative bacteria, *A*. *actinomycetemcomitans*.

From the currently available drugs delivered locally, significant adjunctive reduction of pocket depth and clinical reduction gains were achieved only on MH (gel and capsule) and DOX gel [33]. It is for this reason that we choose MH and DOX to be the comparative antibiotic in this study. Though use of these antibiotics have proven to be an effective treatment modality, problems of antimicrobial resistance to antibiotics have been increasing. On the contrary, high production cost of synthesizing peptides is considered a limitation for using AMPs. Combining AMPs with conventional antibiotics will not only reduce production cost but may also prevent emergence of resistant bacterial strains, reduce toxicity and reduce unwanted side effects of each agent. Furthermore, synergism between the two agents may prove to be effective against already existing resistant bacteria.

In this study, AMPs demonstrated better antimicrobial activity compared to MH and DOX. The higher killing activity of AMPs over DOX may be due to their different mechanism of action. MH and DOX are bacteriostatic agents. Interestingly, at 1 and 5 μM concentrations, MH and DOX stimulated bacterial growth. This finding is similar to the previous report showing increased bacterial growth when sub-inhibitory doses of MH [34] and DOX [35] are given. In contrast, all the peptides showed killing activity even at low concentrations. The mechanism of action of most AMPs against microbes involved disrupting the membrane or forming pores in the membrane resulting in leakage of essential ions and vital constituents such as intracellular nucleotides leading to cell dead [36, 37]. IDR-1018 was originally synthesized for immunomodulatory activity. As such, it has a rather weak direct antimicrobial activity against Gram-negative and Gram-positive bacteria [14]. This was also demonstrated in our experiment wherein IDR-1018 showed the least antimicrobial activity compared to the other peptides. In contrast, LFchimera showed very strong killing ability. This potent antimicrobial effect of LFchimera is in accordance with previous reports indicating strong bactericidal activity against several microorganisms, including Gram-positive and Gram-negative bacteria, fungi and parasites [13, 15, 31, 38–40].

There have been previous studies that demonstrated synergisms between antimicrobial peptides and various antibiotics [41–43]. Because of this, the combination of the DOX and LFchimera was examined to explore possible synergism between agents. This study showed synergism between the two agents with the most synergistic combination demonstrating high killing activity despite an 8-fold reduction of concentration from IC_50_ of both DOX and LFchimera. It may be hypothesized that different mechanism of the agents combined to produce this synergistic result. Destruction of bacterial membrane by LFchimera may have enhanced access of DOX to the bacterial cytoplasm. This phenomena was also suggested in the study of Choi and Lee [43].

In order to initiate disease, bacteria must adhere to the host tissue. *A*. *actinomycetemcomitans* have multiple adhesins including fimbriae, extracellular amorphous material and invasins present on its cell wall [25] that contributes to its virulence. One possible therapeutic approach preventing or treating disease is by interfering bacterial adhesion. In order to explore the possibility of using the agents on anti-adhesion therapy, the drug and peptide alone and in combination was tested on its effect on *A*. *actinomycetemcomitans* adhesion.

Both live and dead bacteria were stained since previous studies have showed that dead bacteria can continue to attach [44] and may contribute to biofilm growth and development [45]. It is notable that even with an increase amount of bacteria to OD_540_ of 0.5 used in the adhesion experiment, the synergistic combination was still able to markedly decrease the amount of adhering bacteria. LFchimera at concentration of 0.125 μM alone had more effect in decreasing adhering bacteria than 1.4 μM DOX. A study by Bolscher et al. [13] have shown that the maximum effect of LFchimera is already reached after 15 min. The 30 min time allotted for bacteria to adhere would have allowed LFchimera to reach its maximum effect and destroy the membrane. From the CLSM micrographs, the samples with LFchimera used alone or in combination have a noticeable increase in red fluorescence compared to the control or DOX alone. From the results we may infer that with the destruction of bacterial membrane, it is possible that the adhesion mechanism of the bacteria has also been destroyed.

Since microorganism in biofilm becomes highly resistant to conventional antibiotic treatment [46], it is important to determine the effect of the antimicrobial agents against *A*. *actinomycetemcomitans* in its biofilm form. Our previous study has reported antibiofilm activity of LFchimera against 1-day old *A*. *actinomycetemcomitans* at 5 μM concentration [16]. This study tested the agents at lower concentrations than previously reported and with the combination of antibiotic DOX. Results of this study revealed that low concentration of LFchimera (0.125 μM) have minimal effect on 1-day old *A*. *actinomycetemcomitans* biofilm in terms of biomass, biofilm thickness, and substratum coverage. Biofilm treated for 15 min in 1.4 μM DOX even showed a slight increase in all measured parameters. On the contrary, the combined agents showed significant decrease in all measured variables which further proves higher activity is achieved when the two agents are combined.

Knowing that bacteria in biofilm are more resistant to antimicrobial agents, the results obtained in the biofilm experiment are expected due to the very low concentrations of DOX (1.4 μM) and LFchimera (0.125 μM) used. Because of this, the combination was further compared with 13 μM DOX and 1.5 μM LFchimera that demonstrated equal killing activity of 66% from the results of the checkerboard experiment. Interestingly, immersion of the 1-day old biofilm in 13 μM DOX further caused a significant overall increase in biomass, biofilm thickness and substratum distribution. The result of 1.4 and 13 μM DOX is similar to a previous study where low concentration of DOX initiated increased biofilm growth [35]. The 1.5 μM LFchimera treated coverslips showed a significant reduction in biomass, average biofilm thickness and substratum coverage when compared to control and 13 μM DOX. This further confirms the antibiofilm activity of LFchimera against *A*. *actinomycetemcomitans* [16] and other Gram-negative bacteria biofilm [47]. The combination of the two agents had the most significant decrease of biomass, biofilm thickness and substratum coverage. This finding suggests that the combined effects of DOX and LFchimera allows disruption of biofilm matrix and bacterial cell membrane by the peptide allowing subsequent penetration of the drug resulting to increased anti-biofilm effect.

In conclusion, this study has proven that LFchimera alone and in combination with DOX exhibited strong antibacterial, anti-adhesion and antibiofilm property against *A*. *actinomycetemcomitans* ATCC 43718. The findings suggest that LFchimera alone or in combination with adjunctive agent should be considered for development as a new potential therapeutic agent that may be used as an adjunctive treatment for periodontitis.
